# Increasing Perceptual Salience Diminishes the Motor Interference Effect From Dangerous Objects

**DOI:** 10.3389/fpsyg.2020.00580

**Published:** 2020-03-27

**Authors:** Rong Cao, Gai Cao, Peng Liu

**Affiliations:** ^1^School of Public Management, Northwest University, Xi’an, China; ^2^The Research Center for Livelihood Security and Social Governance in Shaanxi Province, Xi’an, China

**Keywords:** motor interference effect, motor priming paradigm, perceptual salience, dangerous object, color

## Abstract

Existing research has indicated that dangerous objects may conflict with an individual’s prepared motor actions and thus slow responses. This phenomenon is called the motor interference effect from dangerous objects. However, its origin remains arguable. The current study aimed to preclude an alternative origin and to investigate whether the efficiency of processing a prepared response toward a dangerous object could benefit from increasing the perceptual salience of the object by painting the object red. The design used a shape categorization task to emphasize the dangerous elements of target objects and manipulated target color (gray versus red), target dangerousness (safe versus dangerous) and prime-target congruency (congruent versus incongruent). The null effect of N2 amplitudes between the dangerous and safe conditions precluded the alternative origin and suggested that the motor interference effect did not originate from response inhibition. Furthermore, the results indicated a modulation effect of the motor interference effect in different colors. The classic motor interference effect was observed in the gray target condition, but it diminished in the red target condition. The underlying cognitive processes were reflected in ERPs. More positive P2 and frontal P3 amplitudes were identified in the red target condition than in the gray target condition, which indicated that deeper feature detection was assigned to and more attentional resources were automatically recruited for the red targets than for the gray targets. Analysis of the parietal P3 amplitudes identified a similar result pattern as the mean RTs. A more positive P3 amplitude was identified in the dangerous condition than in the safe condition when the targets were painted gray. In contrast, the P3 amplitudes were identical between the dangerous condition and the safe condition when the targets were painted red. The results indicated that the increased attentional resources facilitated the evaluation of red target dangerousness and thus accelerated reactions to the red dangerous targets; the reaction speeds to those targets were close to those for the reaction speeds to the red safe targets. Detailed processes that underline these components are discussed.

## Introduction

In human life, we constantly interact with objects. We can touch or grasp objects voluntarily, but objects also imply potential action information that might evoke motor responses (called “affordance,” Gibson, 1979). For example, observing a picture of teapot could facilitate an ipsilateral response corresponding to the handle of the teapot (Tucker and Ellis, 1998). Studies have indicated that object properties such as size (Tucker and Ellis, 2001), weight (Brouwer et al., 2006), consistency (Anelli et al., 2010) and dangerousness (Anelli et al., 2013a, b) can influence responses to an object. The current study focuses on modulation of aversive affordance (elicited by dangerous objects) using a motor priming paradigm with a shape categorization task.

The processing of object dangerousness has been investigated from two perspectives. From one perspective, studies have examined the basic processing of dangerous objects when no agent interacts with them. Studies from this perspective have found a threat-superiority effect, suggesting that dangerous stimuli are powerful at capturing attention and that dangerous stimuli are automatically detected (Fox et al., 2000; Öhman et al., 2001; Tipples et al., 2002; Blanchette, 2006). Studies from the other perspective have explored the empathy mechanisms for pain when dangerous objects are presented to an agent. This paradigm allows the investigation of inhibitory mechanisms in pain observation. Studies observed a motor interference effect when participants passively observed other individuals’ pain (Avenanti et al., 2005, 2006; Morrison et al., 2007). For example, key-press responses in Go trials were slower if video clips depicted a scene in which a needle contacted a hand than if they depicted a scene in which a cotton bud contacted a hand (Morrison et al., 2007), suggesting an aversive affordance of dangerous objects. In line with these studies, Anelli et al. (2012) investigated empathy mechanisms with a motor priming paradigm in which a hand prime and a dangerous object were successively presented. The results also indicated a motor interference effect from dangerous objects (responses were delayed in the dangerous target condition compared to the safe target condition), suggesting an aversive affordance that dangerous objects may conflict with an individual’s prepared motor actions and thus slow responses. Moreover, the results identified faster responses in real hand-grasping conditions than in robot hand and real static hand conditions; this suggested that a real grasping hand prime may influence subsequent object responses when it is in a position of potential interaction with target objects. This paradigm is highly significant because it allows the investigation of how individuals control their prepared motor responses when facing an emergent dangerous object. Especially for industrial workers who need to operate machines, dangerous elements (such as saw blades) in machines may cut off their fingers if their motor action is not inhibited in time. Therefore, further investigating the neural mechanisms underlying the paradigm is important and might help reduce the working accident rate.

Subsequent research from our laboratory further investigated the origin of the motor interference effect using a motor priming paradigm combined with a Go/NoGo task (Liu et al., 2017). The experiment used pictures of a left or right hand as primes and dangerous or safe objects on which a green (Go signal) or red (NoGo signal) circle was superimposed as targets. Participants were instructed to prepare for the corresponding key press with the hand that was consistent with the handedness of the prime and not to execute the key press until a Go signal appeared. The experiment aimed to test two candidate origins: (1) the motor interference effect may originate from direct response inhibition elicited by dangerous objects, or (2) the motor interference effect may originate from the evaluation of dangerous objects, and individuals can further decide whether the prepared response should be executed until the encountered dangerousness is analyzed. The first candidate predicted a more negative N2 amplitude [representing conflict detection (Falkenstein et al., 1999)] in the dangerous than in the safe condition. In contrast, the second candidate predicted a more positive parietal P3 amplitude [representing attentional resource assignment (Isreal et al., 1980)] in the dangerous condition than in the safe condition. The results confirmed a classic motor interference effect and further identified a more positive parietal P3 amplitude rather than a more negative N2 amplitude in the dangerous condition than in the safe condition in the Go trials. Therefore, Liu et al. (2017) concluded that the motor interference effect from dangerous objects might originate from danger evaluations.

Nevertheless, an alternative explanation might be responsible for the null effect between dangerous and safe conditions in the N2 component. Specifically, in the Go/NoGo task adopted by Liu et al. (2017), response signals (green or red circles) were presented in the center of the target objects. Participants could narrow their attention at the center of the targets without paying attention to the dangerous elements (dangerous elements such as small shapes with serrated details emerged at the periphery of the dangerous targets) in early processing. Thus, an increase in the N2 amplitude was absent in the dangerous condition. This argument was supported by the results of the P2 amplitudes. Previous studies have indicated that the P2 component reflects the processing of object identification (Viggiano and Kutas, 2000), which emerges at two scalp regions (occipital or frontal areas). The occipital P2 component is suggested to reflect the processing of low-level features of stimuli (e.g., spectral power of visual input) (Martínez et al., 2001; Hansen et al., 2011; De Cesarei et al., 2013). In contrast, the frontal P2 components are suggested to reflect visual feature detection of threats. The frontal P2 amplitude increases as the stimulus becomes more dangerous, which indicates a deeper feature detection of threats in early processing (Carretié et al., 2001; Correll et al., 2006). However, Liu et al.’s (2017) analysis of the frontal P2 amplitudes did not identify a significant difference between dangerous and safe conditions, which suggested that dangerous elements of target objects might not be attended in early processing. Accordingly, the first goal of this study was to clarify whether processing the dangerousness of target objects could enlarge N2 amplitudes. We changed the Go/NoGo task to a shape categorization task (judging the shape of target objects), which forced participants’ attention to the periphery of the target and thus emphasized the dangerousness of target objects.

The second goal of the study was to examine the modulation effect of the motor interference effect by manipulating the perceptual salience of target objects. The results might present a reference for safety management regarding how to increase the efficiency of processing a prepared response toward a dangerous object. Given the conclusion from Liu et al. (2017), a prepared response oriented to a dangerous object is executed after the dangerousness of the object is evaluated. It can be inferred that the efficiency in executing prepared motor readiness toward a dangerous object can be improved by quickly evaluating the dangerousness of objects. Increasing the perceptual salience of dangerous objects might meet this aim because an increase in perceptual salience is shown to increase attentional resources assigned to targets and thus to accelerate the target responses in tasks of emphasizing the perceptual properties of stimuli (Huang and Yeh, 2011). Changing object colors is a typical way to manipulate the perceptual salience of an object. Research in ergonomics and color psychology suggests that longer-wavelength colors such as red and orange are more salient than shorter-wavelength colors such as green and blue (Nakshian, 1964). More direct evidence from Carvalho et al. (2008) indicates that the color red is more salient than the color gray. Accordingly, we hypothesized that relative to painting objects gray, increasing the perceptual salience of objects by painting them red might enable participants to focus their attention on the objects. Therefore, the conflict in processing motor readiness could be resolved more quickly when the dangerous object is red than when it is gray because more attentional resources are recruited to accelerate the danger evaluation of the red target.

To clarify the two issues, the current study used the motor priming paradigm combined with the event-related potential (ERP) technique adopted by Liu et al. (2017) and changed the Go/NoGo task to a shape categorization task. Specifically, a prime and a target were successively presented in each trial on a computer screen. Pictures of a left or right grasping hand were used as primes with the aim to activate left or right motor readiness (Vainio, 2011; Anelli et al., 2012; Vainio et al., 2013). Pictures of smooth gray/red rectangles and circles were used as safe targets. Pictures of shape-matched gray/red rectangular and circular sawblades were used as dangerous targets. The participants were instructed to categorize the target shape and response with the left or right hand as fast as possible. A shape categorization task was performed partly because it may force participants’ attention to the periphery of the target, which may emphasize the dangerousness of the target object. Moreover, this simple task could stimulate a facilitation effect of the red color because a previous study indicated that relative to shorter-wavelength colors, longer-wavelength colors impaired performance on complex tasks but facilitated performance on simple tasks (Type et al., 1998). The design involved manipulating the target color (red versus gray), the target dangerousness (safe versus dangerous), the target shape (rectangular versus round) and the handedness of the prime (left versus right). Note that the handedness of the prime and the target shape composed the prime-target congruency as one independent variable in the data analysis. Because the handedness of the prime may influence the target responses (Vainio, 2011; Vainio et al., 2013), a left- or right-hand prime followed by a left- or right-hand response comprised the congruent condition, which may accelerate target responses. A left- or right-hand prime followed by a right- or left-hand response comprised the incongruent condition, which may delay target responses.

We expected that if the shape categorization task forced participants to attend to the periphery of the targets in early processing, a significant difference between the dangerous and safe conditions should emerge in the frontal P2 component. Specifically, a more positive P2 amplitude should emerge in the dangerous than the safe condition at the frontal area. For the N2 component, if the dangerous objects directly inhibited the prepared responses elicited by the prime, a more negative N2 amplitude should emerge in the dangerous condition than in the safe condition, or if processing of the dangerous objects only recruited more attentional resources for evaluation without inhibiting the prepared response, as suggested by Liu et al. (2017), the difference in the N2 amplitude between the dangerous and safe conditions should be insignificant. Furthermore, we expected similar result patterns between the RTs and the P3 amplitudes because the P3 component is responsible for the motor interference effect. Specifically, a classic motor interference effect, as indicated by a longer RT and a more positive P3 amplitude, should emerge in the dangerous target condition than in the safe target condition when the targets were painted gray because participants would need to recruit more attentional resources to evaluate dangerous targets, which may also delay reactions to those dangerous targets in the gray target condition. In contrast, the color red might recruit more attentional resources to the red targets than to the gray targets. Accordingly, a more positive P3 amplitude should emerge in the red target condition than in the gray target condition. The pre-recruited attentional resources may thereby facilitate the evaluation processes and accelerate reactions to the red dangerous targets similar to reactions to the red safe targets; thus, the motor interference effect might diminish in the red color condition. In a similar vein, the P3 amplitudes for the dangerous target condition should be identical to those for the safe target condition when the targets were painted red because the sufficiently pre-recruited attentional resources might have been assigned to the red targets to evaluate the dangerousness of the red safe and dangerous targets. Additionally, the congruency effect, as indicated by a longer RT in the incongruent condition than in the congruent condition, should emerge in the behavioral results.

## Materials and Methods

### Participants

*A priori* sample size estimation with a large effect size (*f* = 0.4) and 0.95 statistical power was conducted by G^∗^Power to determine the sample size required for the experiment (Faul et al., 2007). The minimum number of participants required for the repeated-measures ANOVA was 9. We then recruited 24 right-handed undergraduate students (ten males) ranging in age from 18 to 25 years (mean = 20.92 years) to obtain more robust results. One participant was excluded from the data analysis because of extremely high myopia. Another was excluded because of a hardware display error. The remaining 22 participants were included in the data analysis (eight males, mean age = 21.05). All of them reported normal or corrected-to-normal visual acuity and reported an absence of neurological disorders. They provided written informed consent and were compensated with 50 yuan RMB. The experiment was performed in compliance with relevant institutional guidelines and was approved by the School of Public Management ethics committee.

### Materials and Apparatus

**FIGURE 1:**
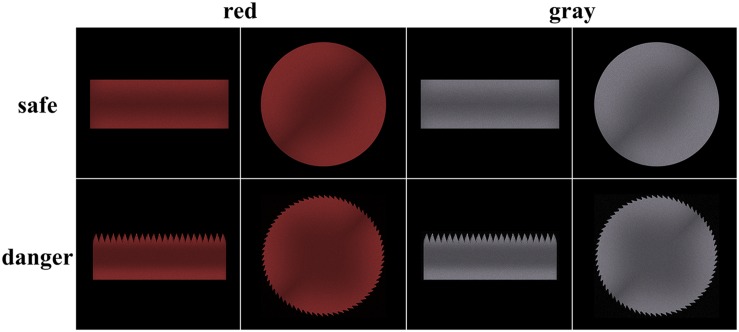
Schematic representation of the target stimuli.

The experimental stimuli included primes and targets. The primes (a left or right hand subtending a visual angle of 13° horizontally and 11° vertically) were identical to those in Liu et al. (2017) to imitate a motor readiness situation. To imitate a spatially matched grasping situation, the left or right hand was presented 2° to the left or right, respectively, of the fixation point. The targets (a visual angle of 10° horizontally and 3.8° vertically for the rectangular targets and a visual angle of 8.8° horizontally and 8.8° vertically for the circular targets) were also identical to those in Liu et al. (2017), with the exception that the targets were painted in red (hue = 0, saturation = 100, brightness = 66) and gray (hue = 0, saturation = 0, brightness = 66) colors (Figure 1). The hue, saturation, and brightness (HSB) color model was used to produce different target colors with matching brightness. The targets were presented in the center of the screen. All stimuli were presented on a black background to reduce eye fatigue. Target dangerousness was assessed by the question “Please evaluate the dangerousness of the target stimuli,” which was answered on a five-point Likert scale (with 1 = not a dangerous object and 5 = an extremely dangerous object) after the main task. Because Likert scales are an ordinal measurement, we performed non-parametric tests for the difference in the assessment scores between the dangerous and safe conditions and between the gray and red color conditions. The results of Wilcoxon signed-rank tests indicated a significant difference between the dangerous and safe conditions (*Z* = −4.12, *p* < 0.001) and a significant difference between the gray and red conditions (*Z* = −2.97, *p* = 0.003). The results indicated that the rectangular saw blade and the circular saw blade were assessed as more dangerous than the smooth rectangle and circle. Moreover, the red targets were assessed as more dangerous than the gray targets.

The experiment was run with E-Prime software (version 2.0, Psychology Software Tools, Inc.) on a standard PC linked to a 17-inch CRT monitor (60-Hz refresh rate). Electroencephalogram (EEG) data were recorded by a NeuroScan system (NeuroScan, Inc.). A Neuroscan Synamp 2 amplifier with a 64 Ag/AgCl electrode cap mounted according to the extended international 10–20 system was used to continuously record EEG data (sampling rate at 500 Hz).

### Procedure

**FIGURE 2:**
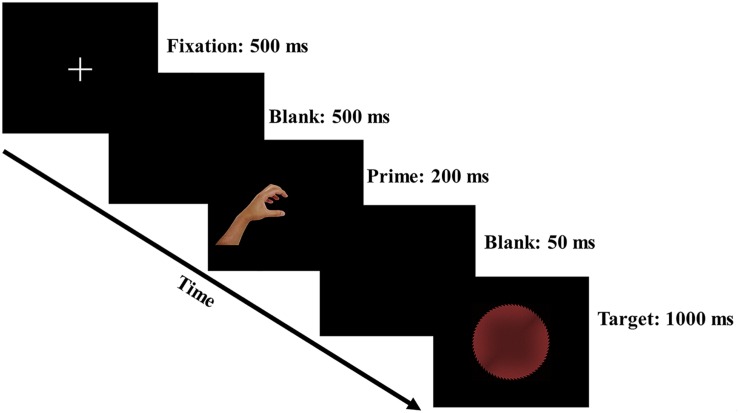
Schematic representation of the trial procedure.

The participants were seated in a dimly lit room with a computer screen placed 60 cm in front of their eyes, and they were instructed to maintain their central eye fixation throughout the experiment. The trial procedure of the experiment is presented in Figure 2. A central fixation cross (500 ms), a blank screen (500 ms), a left- or right-hand prime (200 ms), another blank screen (50 ms) and a target (1,000 ms) were successively presented in each trial. The intertrial interval was randomized within 1,400–1,800 ms to eliminate the expectancy effect for the subsequent trial. The target display was terminated if the response was executed within 1,000 ms. The participants were instructed to respond to the shape of the target as fast as possible on a standard keyboard. Specifically, half of the participants were instructed to use the index finger of their left hand to respond with an F keypress to circular targets (all gray/red safe and dangerous circular targets) and to use the index finger of their right hand to respond with a J keypress to rectangular targets (all gray/red safe and dangerous rectangular targets). To counterbalance the response rule, the other half of the participants were told to use the index finger of their left hand to respond with an F keypress to rectangular targets and to use the index finger of their right hand to respond with a J keypress to circular targets. The response keys were counterbalanced to avoid a possible handedness bias.

The design manipulated the target color (red versus gray), the target dangerousness (safe versus dangerous) and the prime-target congruency (congruent versus incongruent). The experiment consisted of 480 trials, which included 2 types of target color (red versus gray) × 2 types of target dangerousness (safe versus dangerous) × 2 types of prime-target congruency (congruent versus incongruent) × 60 repetitions. Furthermore, the handedness of the prime (left versus right) and the target shape (rectangular versus round) were assigned in equal proportions in each experimental condition. Prior to the formal experiment, a 16-trial practice was conducted, and the formal experiment did not begin until the participant achieved 85% accuracy in the practice phase. The participants were given a break of at least 2 min after every 80 trials and were encouraged to take longer breaks when necessary.

### EEG Recording and Processing

In recording EEG, the signals were bandpass-filtered at 0.05–100 Hz and referenced to the tip of the nose. The electrode impedance was maintained at less than 5 kΩ throughout the experiment. The EEG data were preprocessed using the EEGLAB toolbox (Delorme and Makeig, 2004) according to the following steps: (1) the continuous EEG was high-pass filtered at 0.1 Hz and low-pass filtered at 30 Hz; (2) bad channels were deleted; (3) large drifts and artifacts in the continuous EEG were detected by eye and manually deleted; (4) EEG data contaminated by eye blinks and eye movements were corrected using the independent component analysis (ICA) algorithm (Delorme and Makeig, 2004); (5) the continuous EEG was epoched and time-locked to the target onset in epochs of 3,000 ms with a presplicing point of 1,000 ms, and the epoched data were corrected to the baseline using the 1,000 ms prior to the target onset; (6) the deleted channels were interpolated using the EEGLAB toolbox; (7) the epochs were rereferenced to the bilateral mastoid electrodes; and (8) the epochs with large artifacts (which exceeded ± 100 μV) and incorrect responses were removed. Consequently, the preprocessing rejected 3.5% of the epochs as contaminated across all participants and all conditions.

Before grand-averaging, the artifact-free data were resegmented and initiated from the 200 ms before and the 800 ms after the target onset and were referenced to the baseline (i.e., the 200 ms prior to the target onset). Then, the extracted average waveforms for each participant and condition were used to calculate the grand-average waveforms.

### Statistical Analysis

#### Behavioral Data

Mean RTs and mean error rates for each experimental condition were averaged separately for each participant. RTs for incorrect responses and RTs greater or less than three standard deviations for each participant were excluded from the RT analysis. Before the analysis, Kolmogorov–Smirnov tests of normality were performed on the RTs and error rates for each condition. The results indicated that the distributions of RTs were normal for all conditions (*p*s > 0.32), but the error rates deviated from normality (*p*s < 0.003). Accordingly, the mean RTs and logarithmic correct rates (because 0 cannot be log-transformed, we used correct rates instead of error rates) were analyzed by three-way repeated-measures ANOVAs (Wang et al., 2014). The independent variables were the target color (red versus gray), the target dangerousness (safe versus dangerous) and the prime-target congruency (congruent versus incongruent).

#### ERP Data

**FIGURE 3:**
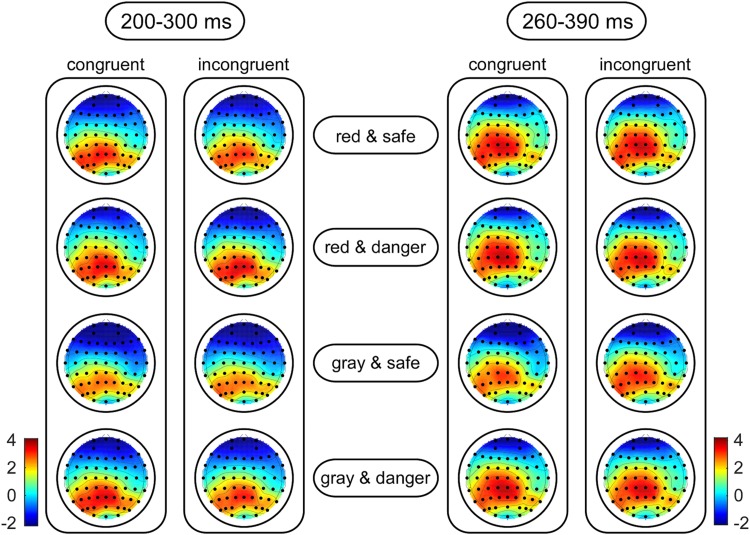
Grand-average topographic plots of the N2 (left panel) and parietal P3 (right panel) components. The scalp topographies of these components are calculated based on the mean amplitude in the 200 to 300-ms time window for the N2 component and in the 260 to 390-ms time window for the parietal P3 component as a function of the target color, the target dangerousness and the prime-target congruency.

For target-locked waveforms, the dependent variables included the peak amplitudes for the P2 (the most positive peak amplitude between 160 and 220 ms) and N2 (the most negative peak amplitude between 200 and 300 ms) components and the mean amplitudes for the frontal P3 (the mean amplitude between 300 and 400 ms) and parietal P3 (the mean amplitude between 260 and 390 ms) components. Kolmogorov–Smirnov tests of normality were also performed on the amplitudes at each condition. The results indicated that the distributions of the amplitudes did not deviate from normality (*p*s > 0.064). Luck (2014) suggested that electrode sites selected for analysis should be those at which the components are large and other components are relatively small so that the measurements of the components of interest are not affected by adjacent components. Accordingly, the N2 and parietal P3 components were analyzed at frontal and centroparietal areas, respectively, where the amplitudes were greatest according to topographical maps (Figure 3). To test whether the dangerousness of targets was identified in early processing, the P2 amplitudes were analyzed at the frontal area (Carretié et al., 2001; Correll et al., 2006), where a clear P2 component was identified (Luck, 2014, p. 315). Moreover, the frontal P3 component (i.e., a classical component representing involuntary attentional processing) was analyzed in the frontal area (Picton, 1992). The independent variables included the target color (red versus gray), the target dangerousness (safe versus dangerous), and the target congruency (congruent versus incongruent). Moreover, channel distributions (left to right) were entered into the analysis as an independent variable to investigate whether an asymmetrical advantage existed in the ERP results (Luck, 2014, p. 314). Accordingly, we selected F3, Fz, and F4 electrodes in the frontal area for analyzing the P2, N2, and frontal P3 components and selected CP3, CPz, and CP4 electrodes in the central-parietal area for analyzing the parietal P3 components. Four-way repeated-measures ANOVAs were used to analyze the effects of the independent variables. The degrees of freedom of the *F*-ratio were corrected using the Greenhouse–Geisser method, and multiple comparisons were adjusted by the Bonferroni method in the analyses. The effect sizes are presented as partial eta-squared values ($\eta_{\text{p}}^{2}$) for the ANOVAs and as *Cohen’s d*s for the *t*-tests.

## Results

### Behavioral Results

**FIGURE 4:**
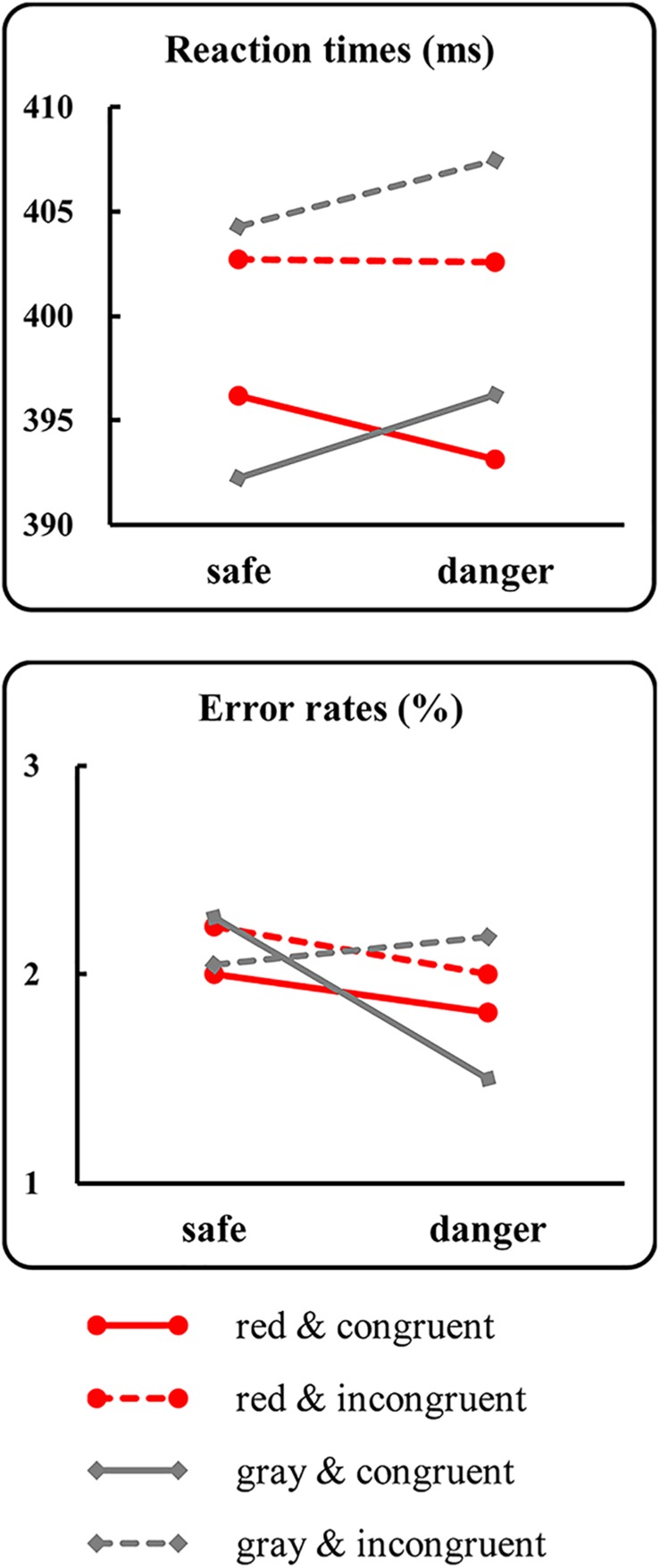
Results of the behavioral tests. The figure presents the mean reaction times (upper panel) and the mean error rates (lower panel) as a function of the target color, the target dangerousness and the prime-target congruency.

For the RTs (Figure 4, line graph), the results identified a significant main effect of prime-target congruency [*F*(1,21) = 10.52, *p* = 0.004, $\eta_{\text{p}}^{2}$ = 0.33]. The *post hoc* analysis indicated that the mean RTs for the incongruent condition (408 ± 63 ms) were longer than those for the congruent condition (400 ± 61 ms). Moreover, a significant two-way interaction between the target color and the target dangerousness [*F*(1,21) = 4.91, *p* = 0.04, $\eta_{\text{p}}^{2}$ = 0.19] was identified. Subsequent paired *t*-tests indicated that the mean RTs for the dangerous condition (402 ± 62 ms) were longer than those for the safe condition [398 ± 59 ms; *t*(21) = 2.31, *p* = 0.03, *Cohen’s d* = 0.48] in the gray color condition. In contrast, the mean RTs for the dangerous condition (398 ± 61 ms) did not significantly differ from those for the safe condition [399 ± 59 ms; *t*(21) = 0.80, *p* = 0.44, *Cohen’s d* = 0.16] in the red color condition. However, analysis of the log-transformed correct rates identified non-significant main effects and interactions (all *p*-values > 0.29). The results of the RTs were more convincing because the mean errors for each condition were less than 2.27%, which may have caused a ceiling effect.

### ERP Results

**FIGURE 5:**
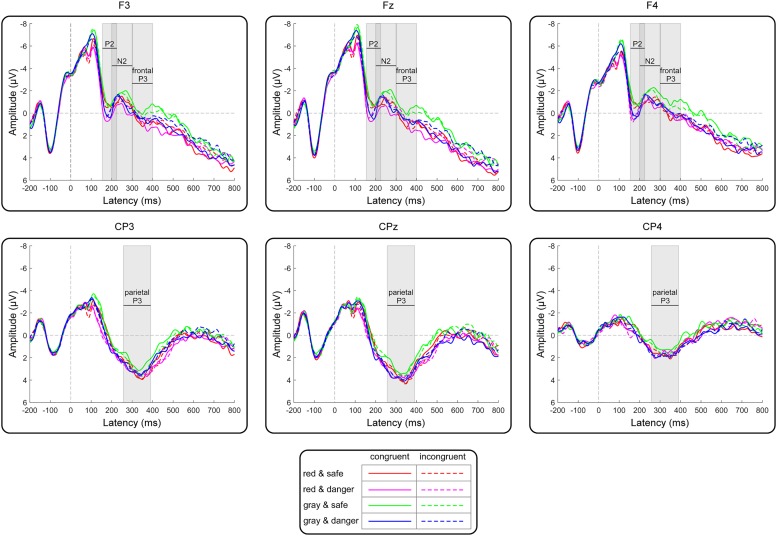
Grand-average target-locked ERPs for the frontal (F3, Fz, and F4) and central-parietal (CP3, CPz, and CP4) electrodes as a function of the target color, the target dangerousness and the prime-target congruency. The gray rectangles indicate the analyzed time windows for the P2, N2, frontal P3, and parietal P3 components.

Grand averages of target-locked ERPs are presented in Figure 5. Analysis of the P2 peak amplitudes revealed a significant main effect of the target color [*F*(1,21) = 5.77, *p* = 0.03, $\eta_{\text{p}}^{2}$ = 0.22]. The *post hoc* analysis indicated that the P2 amplitude for the red target condition (1.43 ± 0.64 μV) was more positive than that for the gray target condition (0.99 ± 0.56 μV). Moreover, a significant main effect of target dangerousness was identified [*F*(1,21) = 8.90, *p* = 0.007, $\eta_{\text{p}}^{2}$ = 0.30]. The *post hoc* analysis indicated that the P2 amplitude for the dangerous target condition (1.74 ± 0.66 μV) was more positive than that for the safe target condition (0.69 ± 0.57 μV). Analysis of the N2 peak amplitudes revealed that none of the main effects or interactions reached significance (all *p*-values > 0.11) (Table 1).

**TABLE 1:** ANOVA results (*F*-values, *p*-values, and partial eta-squared values) of the amplitudes of the P2, N2, frontal P3, and central-parietal P3 components as a function of the area (channel distributions), the target color, the target dangerousness and the prime-target congruency.

Factors	*df*	P2 amplitudes			N2 amplitudes			Frontal P3 amplitudes			Central-parietal P3 amplitudes		
		*F*	*p*	$\eta_{\text{p}}^{2}$	*F*	*p*	$\eta_{\text{p}}^{2}$	*F*	*p*	$\eta_{\text{p}}^{2}$	*F*	*p*	$\eta_{\text{p}}^{2}$
Area	2, 42	0.03	0.86	0.002	0.04	0.95	0.002	1.89	0.16	0.08	13.79	0.001*	0.40
Target color	1, 21	5.77	0.03*	0.22	1.77	0.20	0.08	8.68	0.008*	0.29	2.37	0.14	0.10
Target dangerousness	1, 21	8.90	0.007*	0.30	2.75	0.11	0.12	0.92	0.35	0.04	2.86	0.11	0.12
Prime-target congruency	1, 21	0.001	0.98	0.001	0.004	0.95	0.001	0.01	0.92	0.001	0.03	0.86	0.002
Area × Target color	2, 42	0.007	0.93	0.001	0.39	0.67	0.02	0.43	0.62	0.02	2.63	0.09	0.11
Area × Target dangerousness	2, 42	1.02	0.32	0.05	1.58	0.22	0.07	0.20	0.76	0.01	0.46	0.58	0.02
Target color × Target dangerousness	1, 21	0.04	0.85	0.002	0.49	0.49	0.02	5.19	0.03*	0.20	4.46	0.05*	0.18
Area × Target color × Target dangerousness	2, 42	0.06	0.81	0.003	2.06	0.16	0.09	0.58	0.50	0.03	0.94	0.38	0.04
Area × Prime-target congruency	2, 42	0.92	0.35	0.04	1.10	0.34	0.05	2.91	0.09	0.12	0.15	0.83	0.007
Target color × Prime-target congruency	1, 21	0.002	0.97	0.001	0.04	0.84	0.002	2.01	0.17	0.09	0.44	0.52	0.02
Area × Target color × Prime-target congruency	2, 42	0.09	0.76	0.004	1.07	0.35	0.05	0.28	0.73	0.01	0.62	0.51	0.03
Target dangerousness × Prime-target congruency	1, 21	1.73	0.20	0.08	0.04	0.85	0.002	6.50	0.02*	0.24	1.39	0.25	0.06
Area × Target dangerousness × Prime-target congruency	2, 42	0.08	0.78	0.004	0.01	0.98	0.001	1.32	0.28	0.06	0.42	0.62	0.02
Target color × Target dangerousness × Prime-target congruency	1, 21	0.06	0.82	0.003	0.18	0.68	0.008	0.01	0.94	0.001	0.21	0.65	0.01
Area × Target color × Target dangerousness	2, 42	0.190	0.67	0.009	0.02	0.96	0.001	0.96	0.39	0.04	0.60	0.55	0.03

df = degrees of freedom. *p ≤ 0.05.

Analysis of the frontal P3 amplitudes revealed a significant main effect of color [*F*(1,21) = 8.68, *p* = 0.008, $\eta_{\text{p}}^{2}$ = 0.29]. The *post hoc* analysis indicated that the frontal P3 amplitude for the red target condition (0.46 ± 1.05 μV) was more positive than that for the gray target condition (−0.05 ± 0.97 μV). Moreover, the two-way interaction between the target color and the target dangerousness was significant [*F*(1,21) = 5.19, *p* = 0.03, $\eta_{\text{p}}^{2}$ = 0.20]. Subsequent paired *t*-tests indicated that the frontal P3 amplitude for the dangerous condition (0.26 ± 4.53 μV) was more positive than that for the safe condition [−0.37 ± 4.64 μV; *t*(21) = 2.35, *p* = 0.03, *Cohen’s d* = 0.50] in the gray color condition. In contrast, the frontal P3 amplitude for the dangerous condition (0.36 ± 4.76 μV) did not significantly differ from that for the safe condition [0.56 ± 5.14 μV; *t*(21) = 0.63, *p* = 0.53, *Cohen’s d* = 0.14] in the red color condition. Additionally, the results identified a significant two-way interaction between target dangerousness and prime-target congruency [*F*(1,21) = 6.50, *p* = 0.02, $\eta_{\text{p}}^{2}$ = 0.24]. However, subsequent paired *t*-tests indicated only a nearly significant difference between the dangerous and safe conditions in the congruent condition [*t*(21) = 1.95, *p* = 0.06, *Cohen’s d* = 0.43] and a non-significant difference between the dangerous and safe conditions in the incongruent condition [*t*(21) = 0.28, *p* = 0.78, *Cohen’s d* = 0.07].

Analysis of the parietal P3 amplitudes revealed a significant main effect of the channel distribution [*F*(2,42) = 13.79, *p* < 0.001, $\eta_{\text{p}}^{2}$ = 0.40]. The *post hoc* analysis indicated that the parietal P3 amplitudes for the left (3.09 ± 0.91 μV) and middle (3.47 ± 1.09 μV) electrodes were more positive than that for the right (1.59 ± 1.11 μV) electrode. Moreover, a significant two-way interaction between the target color and the target dangerousness was identified [*F*(1,21) = 4.46, *p* = 0.05, $\eta_{\text{p}}^{2}$ = 0.18]. Subsequent paired *t*-tests indicated that the parietal P3 amplitude for the dangerous condition (2.87 ± 4.82 μV) was more positive than that for the safe condition [2.32 ± 4.66 μV; *t*(21) = 2.34, *p* = 0.03, *Cohen’s d* = 0.50] in the gray target condition. In contrast, the parietal P3 amplitude for the dangerous condition (2.84 ± 4.88 μV) did not significantly differ from that for the safe condition [2.83 ± 4.84 μV; *t*(21) = 0.07, *p* = 0.95, *Cohen’s d* = 0.01] in the red target condition.

## Discussion

The present study had two aims. The first aim was to preclude an alternative explanation for Liu et al. (2017) that the null effect of the N2 amplitudes between the dangerous and safe conditions emerged because participants narrowed their attention to the centrally presented Go/NoGo signals without paying attention to the peripherally presented dangerous elements (small shapes with serrated details) in early processing. Accordingly, in the current design, the Go/NoGo task was changed to a shape categorization task, which forced participants’ attention to the periphery of the target and thus emphasized the dangerous elements of the target objects. The second aim was to investigate the modulation effect of the motor interference effect by manipulating the perceptual salience of the target objects. We increased the perceptual salience of the targets by painting them red (versus gray) and further manipulated the dangerousness of the targets (safe versus dangerous) and the prime-target congruency (congruent versus incongruent). We hypothesized that painting objects red might pre-recruit more attentional resources to the objects and that these resources might facilitate the evaluation processes and accelerate reactions to the red dangerous targets similar to those to the red safe targets; thus, the motor interference effect might diminish in the red color condition.

The behavioral results identified a classic motor interference effect, which was evidenced by a longer RT for the dangerous target condition than for the safe target condition when the targets were painted gray. In contrast, the motor interference effect diminished in the red target condition, which was evidenced by a non-significant RT difference between the safe and dangerous conditions. The behavioral results support the hypothesis and suggest that relative to painting the target gray, painting the target red diminishes the RT difference in responding to safe and dangerous targets. However, two reasons might explain the diminished RT difference: (1) the RTs were accelerated in the dangerous target condition when the targets were red compared to when they were gray, or (2) the RTs were delayed in the safe target condition when the targets were red compared to when they were gray. To distinguish between these two possibilities, differences in the mean RTs between the red and gray conditions were separately tested in the safe and dangerous conditions via paired *t*-tests. The results identified a non-significant difference between the gray and red target conditions when the targets were safe [*t*(21) = 0.65, *p* = 0.53, *Cohen’s d* = 0.13]. However, the mean RT for the dangerous targets was slightly shorter (nearly significant) in the red color condition than in the gray color condition [*t*(21) = 1.91, *p* = 0.07, *Cohen’s d* = 0.39]. Although the result of the paired *t*-test was marginally significant, the results supported the first possibility and suggested that the RTs were slightly accelerated in the dangerous target condition when the targets were red compared to when they were gray. Additionally, the behavioral results identified a classic congruency effect, which was evidenced by longer RTs for the incongruent condition than for the congruent condition. The results suggested that processing the left- or right-hand primes influenced the target responses. This finding of a significant congruency effect contradicts an argument that target processing is not under motor readiness status because the left- or right-hand prime, which aims to activate response readiness, is not related to the target response; thus, participants might ignore the prime. If this argument holds true, the target responses could not be accelerated in the congruent condition compared to the incongruent condition. Obviously, the significant congruency effect contradicts this argument and suggests that target responses are accelerated in the congruent condition compared to the incongruent condition, although the response rules are not related to the primes.

The ERP results further clarified the underlying cognitive processes. Analysis of the P2 component identified a more positive P2 amplitude for the dangerous condition than for the safe condition at the frontal area. Moreover, a more positive frontal P2 amplitude was identified for the red target condition than for the gray target condition. As stated in the introduction, the P2 component reflects the processing of object identification (Viggiano and Kutas, 2000), with the occipital P2 component reflecting the processing of low-level features of stimuli (Martínez et al., 2001; Hansen et al., 2011; De Cesarei et al., 2013) and the frontal P2 component reflecting visual feature detection of threats. Deeper feature detection increases the frontal P2 amplitudes accordingly (Carretié et al., 2001; Correll et al., 2006). The results of the frontal P2 amplitudes suggest that target features such as target dangerousness and target color may attract attention in early processing. Deeper feature detection is involved in processing dangerous targets and red targets because participants detect threats in these objects. The results of the frontal P2 amplitudes were consistent with the results of the dangerousness assessment task via self-report, which also identified a more dangerous perception of the dangerous targets than the safe targets and a more dangerous perception of the red targets than the gray targets. Regarding the N2 component, non-significant main effects and interactions of the N2 amplitudes were consistent with the findings of Liu et al. (2017), which suggest that prime-elicited response readiness is not directly suppressed by dangerous targets and that the observed behavioral motor interference effect in the gray color condition does not originate from response inhibition. Importantly, the results of the P2 and N2 amplitudes clarified the first aim of the study. A more positive frontal P2 amplitude in the dangerous condition than in the safe condition suggests that target features of dangerousness are fully perceived in the early processing of targets. The results suggest that relative to a Go/NoGo task, a shape categorization task does force participants’ attention to the periphery of the targets and thus emphasizes the dangerousness of the target objects. However, analysis of the N2 amplitudes still identified a null effect between the dangerous and safe conditions, similar to that reported by Liu et al. (2017); thus, the findings confirm the conclusion of Liu et al. (2017) that the motor interference effect does not originate from response inhibition, even though dangerous elements are fully perceived in early processing.

The frontal P3 (i.e., P3a) is a stimulus-driven component that originates from frontal attention mechanisms (Polich and Criado, 2006; Polich, 2007). This component is supposed to reflect involuntary attentional processing (Polich, 2003). The results identified a more positive frontal P3 amplitude in the red target condition than in the gray target condition, which supports the hypothesis and indicates that red targets automatically recruit more attentional resources than gray targets. Moreover, a significant interaction between target color and target dangerousness was identified. Subsequent analysis identified a more positive frontal P3 amplitude for the dangerous target condition than for the safe target condition when the targets were painted gray. In contrast, the frontal P3 amplitudes were identical between the dangerous and safe target conditions when the targets were painted red. The results indicate that more attentional resources are automatically assigned to dangerous targets than to safe targets when the targets are painted gray.

The parietal P3 (i.e., P3b or P300) component maximally emerged in central-parietal areas (Picton, 1992), and its amplitude was larger when participants exerted more effort in a task; this leads to the suggestion that the parietal P3 amplitude can be used as a measure of voluntary attentional resource allocation to process task-relevant events (Isreal et al., 1980; Picton, 1992; Volpe et al., 2007; Luck, 2014). Analysis of the parietal P3 amplitudes also identified a significant two-way interaction between target color and target dangerousness. Subsequent analysis identified a more positive P3 amplitude for the dangerous target condition than for the safe target condition when the targets were painted gray. In contrast, the P3 amplitudes were identical between the dangerous and safe target conditions when the targets were painted red. The results of parietal P3 amplitudes suggested that target dangerousness was differentially processed in the gray and red target conditions. In the gray target condition, participants recruited more attentional resources to evaluate the dangerous target than to evaluate the safe target. In the red color condition, however, participants automatically recruited more attentional resources for the red targets than for the gray targets, as reflected by the frontal P3 component, and the sufficiently pre-recruited attentional resources were assigned to the red targets to evaluate the dangerousness of both the safe and dangerous red targets. Accordingly, the parietal P3 amplitudes for the dangerous target condition were identical to those for the safe target condition when the targets were painted red.

Morrison et al. (2007) suggests that approach-type button presses (executing button presses) and withdrawal-type button releases (executing button releases) are differentially affected by inhibitory and facilitatory mechanisms. The former response pattern may slow participants’ responses to dangerous objects and elicit a motor interference effect, while the latter may accelerate participants’ responses to dangerous objects. Although the current study focused on the modulation effect of the motor interference effect, investigating the facilitatory mechanisms is also important because they reflect the aversive affordance of dangerous objects. A Go/NoGo task might be suitable for investigating facilitatory mechanisms (pressing a button in a Go trial and releasing the button in the next Go trial) in further investigations.

## Conclusion

In summary, the current study first confirmed that the origin of the motor interference effect does not originate from direct response inhibition from dangerous objects, as indicated by a null effect between the dangerous and safe conditions in the N2 amplitudes. Furthermore, this study investigated whether processing a prepared response toward a dangerous object could benefit from increasing the perceptual salience of the object by painting the dangerous object red. Both the behavioral and ERP results indicated that the participants were sensitive to the color of the targets and that target dangerousness was differentially processed in the gray and red target conditions. In the gray target condition, a classic motor interference effect, as indicated by faster responses and larger P3 amplitudes in the dangerous condition than in the safe condition, was identified. In the red target condition, deeper feature detection was assigned to the red targets than to the gray targets, as reflected by the P2 amplitudes in early processing. Thus, more attentional resources were automatically recruited for the red targets than for the gray targets, which was evidenced by the frontal P3 amplitudes. The pre-recruited attentional resources facilitated the evaluation of dangerousness and thus accelerated reactions to the red dangerous targets, reaching reaction speeds similar to those of the red safe targets. Accordingly, RTs for the red dangerous condition were identical to those for the red safe condition. Moreover, sufficient attentional resources were recruited to evaluate the dangerousness of the safe and dangerous targets when they were painted red. Therefore, parietal P3 amplitudes for the red dangerous target condition were identical to those for the red safe target condition. The practical value of this study is that it might provide a reference for safety management by showing that increasing the perceptual salience of a dangerous object is useful for increasing efficiency in processing a prepared response toward that dangerous object. For example, painting the body of a machine in gray and painting dangerous elements of the machine in red could help operators quickly attend to dangerous elements and react to them faster.

## Data Availability Statement

The datasets generated for this study are available on request to the corresponding author.

## Ethics Statement

The studies involving human participants were reviewed and approved by School of Public Management ethics committee. The patients/participants provided their written informed consent to participate in this study.

## Author Contributions

PL designed the experiment, and created the stimulus materials and analyzed the data. GC and PL collected the data. All authors contributed to the writing process and agreed on the final text.

## Conflict of Interest

The authors declare that the research was conducted in the absence of any commercial or financial relationships that could be construed as a potential conflict of interest.
